# Effect of Pei Yuan Tong Nao capsules on cerebral infarction: A protocol for systematic review and meta-analysis

**DOI:** 10.1097/MD.0000000000029050

**Published:** 2022-03-18

**Authors:** Wangkun Chen, Junchi Yu, Suting Li

**Affiliations:** ^a^ *Department of Encephalopathy, Wuxi No. 2 Chinese Medicine Hospital, Jiangsu, China,* ^b^ *Department of Internal Medicine, BinZhou Polytechnic, Shandong, China.*

**Keywords:** cerebral infarction, function recovery, meta-analysis, Pei Yuan Tong Nao

## Abstract

**Background:**

Pei Yuan Tong Nao (PYTN) capsules has been widely used for the treatment of cerebrovascular disease, including cerebral infarction. However, the mechanisms of action of PYTN capsule on cerebral infarction are ambiguous and unclear. We conducted a protocol for systematic review and meta-analysis to evaluate the efficacy and safety of PYTN capsules for the treatment of cerebral infarction.

**Methods:**

A comprehensive search of several databases from 1966 to February 2022 will be conducted. The databases includes Ovid Medline In-Process & Other NonIndexed Citations, Ovid MEDLINE, Ovid EMBASE, Ovid PsycINFO, Ovid Cochrane Central Register of Controlled Trials, Ovid Cochrane Database of Systematic Reviews, and Scopus. Two authors independently performed the literature searching, data extraction, and quality evaluation. The risk of bias in each included study will be assessed utilizing the Cochrane Collaboration’s risk of bias tool. The Review Manager 5.3 (Cochrane Collaboration, Oxford, UK) will be used to analyze the data.

**Result:**

A synthesis of current evidence of PYTN capsules for cerebral infarction will be provided in this protocol.

**Conclusion:**

This review will provide more reliable references to help clinicians make decisions when dealing with cerebral infarction.

## 1. Introduction

Stroke is the first cause of death among Chinese residents with over 2 million new cases annually, of which ischemic stroke accounts for 70%.^[1,2]^ Ischemic stroke is also known as cerebral infarction, which is the ischemic necrosis or cerebral softening of local brain tissues caused by the obstruction of acute local blood supply in brain tissues, ischemia, and hypoxia.^[3,4]^ Cerebral infarction is more common in the elderly, which has a high disability rate and high fatality rate. Even after treatment, the patients may still have cognitive and physical dysfunction with different residual levels, their health and quality of life are seriously threatened by the disease.^[5,6]^

Traditional Chinese medicine (TCM) has been used in the treatment and prevention of ischemia throughout China.^[7,8]^ TCM regimen most often includes a mixed composition of herbs given in a specific ratio, which was fine-tuned by centuries-old empirical practice. As against the western medicinal practice of giving a drug for each indication, TCM has multiple roles, thereby eliminating the need to take multiple medications. Further, since most of the medicines are given as a combination of multiple drugs, the repertoire of the clinical indications for each drug combination also increases.^[9]^ Different TCM herbs have anti-inflammatory and antioxidant properties,^[10]^ cause vasodilation, increase cerebral blood flow velocity, inhibit platelet aggregation, protect against reperfusion injury, and increase tissue tolerance to hypoxia.

Pei Yuan Tong Nao (PYTN) capsules consist of 14 different herbal ingredients that replenish the kidneys, which in TCM terminology means that they are potentially beneficial for any vascular “syndrome” including cerebral infarction.^[11]^ At present, the treatment options for cerebral infarction in western medicine are limited and scientific evidence of TCM for its treatment is scarce. Therefore, we conducted a protocol for systematic review and meta-analysis to evaluate the efficacy and safety of PYTN capsules for the treatment of cerebral infarction.

## 2. Material and methods

This protocol is reported following the preferred reporting items for systematic reviews and meta-analysis protocols (PRISMA-P) statement guidelines.^[12]^ We have registered this study at Open Science Framework (OSF, https://osf.io/). The registration DOI of this study is 10.17605/OSF.IO/RMN49. If there are any changes, we will update the changes in our full review. Ethical approval is not required for this study since it relies on secondary data.

### 2.1. Inclusion criteria

#### 2.1.1. Study type.

In this work, we will include randomized controlled trials of PYTN capsules of any size and duration in adult populations ( ≥ 18 years). NonRCTs and observational study will be excluded. Studies published in English and Chinese will be included.

#### 2.1.2. Types of patients.

This study will include patients diagnosed with cerebral infarction by head computed tomography/magnetic resonance imaging. Included patients had no restrictions on age, sex, economic status, severity of the disease, or education.

#### 2.1.3. Intervention type.

Studies in which interventions in-volved PYTN capsules alone or combined with other routine pharmacotherapies will be included. In the control group, interventions will include placebo or conventional pharmacotherapies recommended by guidelines. Studies with different conventional pharmacotherapies in the control and treatment groups will be excluded.

#### 2.1.4. Outcomes.

The criterion of therapeutic efficiency on neurological functions and daily living activities will be assessed by the National Institute of Health Stroke Scale, modified Rankin Scale, activities of daily living, and Barthel Index (BI). The cognitive functions will be evaluated by minimental state examination and Montreal Cognitive Assessment.

### 2.2. Search methods

A comprehensive search of several databases from 1966 to February 2022 will be conducted. The databases includes Ovid Medline In-Process & Other NonIndexed Citations, Ovid MEDLINE, Ovid EMBASE, Ovid PsycINFO, Ovid Cochrane Central Register of Controlled Trials, Ovid Cochrane Database of Systematic Reviews, and Scopus. Two authors will independently draft and carry out the search strategy. In addition, we manually retrieve other resources, including the reference lists of identified publications, conference articles, and gray literature. The key terms used for the search are “Pei Yuan Tong Nao,” “cerebral infarction” and “randomized controlled trial”. The retrieval process will be presented in Figure 1.

**Figure:**
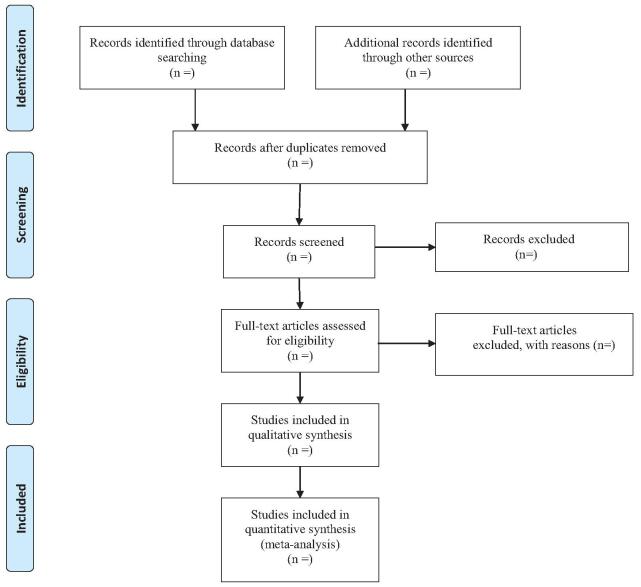
**Figure 1.** Flow diagram of study selection.

### 2.3. Data extraction

We will extract and record the first author’s name, year of publication, study design, group information, age, gender, dropouts, sample size, duration of intervention, outcomes, and adverse effects from the studies that met the inclusion criteria. We will contact the corresponding authors for additional information if necessary.

### 2.4. Risk of bias assessment

The risk of bias in each included study will be assessed utilizing the Cochrane Collaboration’s risk of bias tool.^[13]^ Two researchers will independently evaluate the bias based on the following items: random sequence generation, allocation concealment, blinding of the participants and personnel, blinding of the outcome assessments, incomplete outcome data, selective reporting, and other sources of bias. The studies will be evaluated as low risk, high risk, and unclear risk. Inconsistencies will be resolved by discussion with other reviewers.

### 2.5. Data analysis

The Review Manager 5.3 (Cochrane Collaboration, Oxford, UK) will be used to analyze the data. For outcomes, we will use relative risk and 95% confidence interval to evaluate dichotomous outcomes, while using standardized mean difference with 95% confidence interval to assess continuous variables. The heterogeneity between RCTs will be calculated by Cochrane χ^2^ and *I*^2^ tests. If *P* ≥ .05 and *I*^2^ ≤ 50%, no statistical heterogeneity is observed, the data will be calculated with a fixed-effect model. If *P* < .05 and *I*^2^ > 50%, the random effect model will be used. If there is significant heterogeneity, subgroup analysis will be conducted based on different interventions, controls, durations of treatment, and outcome measures. We will carry out sensitivity analyses to investigate the robustness of the study conclusions. In this way, we will be able to assess the impact of low-quality studies on the overall results and whether the results are robust.

### 2.6. Assessment of publication biases

If there are more than 10 studies included, a funnel plot analysis will be drawn to assess the publication bias and Egger test in Stata 14.0 (Stata Corp, College Station, TX) will be conducted for statistical investigation.

### 2.7. Assessment of quality of evidence

We will use the Grading of Recommendations Assessment, Development, and Evaluation (GRADE) to assess the results. In the GRADE system, the quality of evidence will be categorized into 4 levels: high, moderate, low, and very low quality.

## 3. Discussion

As the pace of population aging accelerates in China, the incidence of cerebral infarction is increasing rapidly, imposing a major economic burden on the family and society.^[14,15]^ According to the theory of Chinese medicine, “blood stasis and stagnation” is regarded as the core pathogenesis of cerebral infarction. Therefore, promoting blood circulation and removing blood stasis is a vital method for the treatment of cerebral infarction.^[16]^ PYTN capsules, a TCM that has been approved for stroke treatment in clinical uses, consists of Polygoni Multiflori Radix (zhiheshouwu), Rehmanniae Radix Praeparata (shudihuang), Asparagi Radix (tiandong), Testudinis Carapaxet Plastrum (guijia), Cervi Cornu Pantotrichum (lurong), Cistanches Herba (roucongrong), Cinnanmomi Cortex (rougui), Radix Paeoniae Rubra (chishao), Scorpio (quanxie), Hirudo (shuizhi), Lumbricus (qiuyin), Crataegi Fructus (shanzha), Poria (fuling), and Glycyrrhizae Radixet Rhizoma (zhigancao).^[17]^ However, the mechanisms of action of PYTN capsule on cerebral infarction are ambiguous and unclear. Therefore, there is a need to clarify and illustrate the function and action modes of PYTN capsule on targets involved in cerebral infarction. We hope that the result of this review will provide more reliable references to help clinicians make decisions when dealing with cerebral infarction.

## Author contributions

**Methodology:** Junchi Yu.

**Writing** - **original draft:** Wangkun Chen.

**Writing** - **review & editing:** Suting Li.
